# Improvement of safflower oil quality for biodiesel production by integrated application of PGPR under reduced amount of NP fertilizers

**DOI:** 10.1371/journal.pone.0201738

**Published:** 2018-08-10

**Authors:** Asia Nosheen, Rabia Naz, Ayesha T. Tahir, Humaira Yasmin, Rumana Keyani, Blagoj Mitrevski, Asghari Bano, Sung Tong Chin, Philip John Marriott, Ishtiaq Hussain

**Affiliations:** 1 Department of Biosciences, COMSATS University, Chak Shahzad, Islamabad, Pakistan; 2 Australian Centre for Research on Separation Science, School of Chemistry, Monash University, Clayton, VIC, Australia; 3 Department of Biosciences, Faculty of Basic Sciences, University of Wah, Pakistan; 4 Department of Agriculture, Gilgit-Baltistan, Pakistan; Institute for Sustainable Plant Protection, C.N.R., ITALY

## Abstract

Safflower is an important industrial oil seed and bioenergy crop in semi-arid subtropical regions due to its potential to grow on marginal land and having good percentage of seed oil contents which is an important parameter for biofuel production. However, it is an ignored crop in Pakistan. In order to improve the crop productivity and reduce the use of agrochemicals for sustainable biodiesel feedstock production, an experiment was conducted for two years to improve the fatty acid composition and oil quality of *Carthamus tinctorius* L. (safflower) by the inoculation of *Azospirillum* and *Azotobacter* alone as well as in combined application with nitrogen and phosphate (NP) fertilizers on cultivars Thori and Saif-32 under field conditions. Separation and quantification of fatty acids were done on precise comprehensive two-dimensional gas chromatography (GC×GC). The results showed that fatty acid profile specifically monounsaturated fatty acids i-e oleic acid (C18:1) was significantly improved by *Azospirillum* supplemented with the quarter dose of NP fertilizers (SPQ) with concomitant decrease in polyunsaturated fatty acids by the respective treatment. Oil quality attributes such as acid value, saponification number, iodine value, refractive index and free fatty acid contents were reduced by the application of *Azotobacter* and *Azospirillum* in combination with half and quarter doses of NP fertilizers treatments (BTH, SPH, BTQ and SPQ). The reduction in these variables is positively linked with improved biodiesel yield and quality. It can be concluded that application of *Azospirillum* and *Azotobacter* not only reduced the use of NP fertilizers up to 50%–75% but also improved the oil quality in order to obtain environment friendly, sustainable and green fuel.

## Introduction

Due to increased growth of population and change in life style, the consumption of energy has been increased in these days. This increase in the demand of energy, limiting the resources of fossil fuel and rising the prices of oil internationally have renewed the interest towards the search of alternative sources of energy (fuels) [1]. In addition, there is an increasing problem of global warming, accumulation of greenhouse gases and environmental pollution leading to climate change which in turn influences the crop productivity. The search for the renewable, sustainable and environmental friendly energy resources is the key factor for improving the economy of a country and reducing the emission of greenhouse gases. Therefore, in order to meet the energy demands, each country is focusing to search the alternative energy sources and among those, biofuel (i-e biodiesel) is one option. [2]. However, main barrier for the production of biodiesel is competition with use of oil for food. Measures which can be adopted to overcome this conflict are to use non edible oil resources, to increase production using waste land areas and to improve the productivity of crops by various measures such as use of chemical and biofertilizers (Plant Growth Promoting Rhizobacteria: PGPR).

Due to incessant use of chemical fertilizers, soil ecology has been subverted which resulted in many environmental problems such as degradation of soil fertility status, soil toxicity, contamination of ground water which leads towards the adverse effects on environment as well as on human [3].

The amalgamation of bio-fertilizers and chemicals is one of the essential measures for the production of sustainable agriculture [4]. Biofertilizer is the term used for fertilizers which are composed up of living microorganism and enhance the growth and productivity of plant by various mechanisms when applied to the rhizospheric soil or the seed of the plant. For sustainable and eco-friendly farming ecosystem, the application of biofertilizers is an appropriate option which helps to reduce the environmental problems due to a considerable extent. [5]. The use of PGPR to enhance plant growth and yield and improve soil health is an emerging trend in present day agriculture. Application of combinations of PGPR and chemical fertilizer improved the yield traits, grain yield, and production of dry matter of barley crop by direct or indirect mechanisms [6].

*Carthamus tinctorius* L. (safflower) is among the important oil seed crops and it is adapted for salt and drought stress conditions. Principally, it is cultivated for the oil used both for food and industrial implications and it is a potential source of biodiesel. As safflower is composed up of complex mixture of unsaturated fatty acids and the separation and quantification of those fatty acids on one dimensional gas chromatography was not satisfactory. Therefore, the use of comprehensive two-dimensional chromatograms containing many peaks and higher peak capacities [7] improves analyses. Another advantage is the formation of two dimensional chromatogram of chemically similar compound which can help for the identification of unknown compounds [8].

A part of this study containing agronomic, biomass, biochemical and yield data of safflower in response to *Azospirillum*, *Azotobacter* and NP fertilizers and the method developed for fatty acid analysis by comprehensive two-dimensional chromatography (GC×GC) has already been published [9, 10]. This part of the study was aimed to improve the oil quality of substrate (i-e safflower) used as a feedstock for biodiesel production using ecofriendly sustainable measures by altering the composition of fatty acids as well as refining oil properties that directly affect the biodiesel quality. Two cultivars of safflower were selected to know their performance under provided conditions.

## Materials and methods

Under the natural conditions, an experiment was conducted on field scale in 2011 and 2012 at the Department of Plant Sciences, Quaid-i-Azam University. Experimental design was a randomized complete block design (RCBD) containing three replicates and the plot size for each treatment was of 1×1 m^2^. The distance between the rows was 45 cm. The certified seeds of both varieties of safflower (cultivars Thori and Saif-32) were provided by the National Agricultural Research Centre (NARC). Prior to sowing, the seeds were surface sterilized with 95% ethanol and washed consecutively for three to four times with distilled autoclaved water.

### Method of seed inoculation

The PGPRs (*Azotobacter vinelandii* and *Azospirillum brasilense*) were applied as seed inoculation at the rate of 10^6^ bacterial cells/seed. To prepare inoculum, 100 mL of Luria Bertani media (LB) was inoculated with the cultures of *Azospirillum brasilense* and *Azotobacter vinelandii* (24 h old) and kept on shaker (Excella E24, USA) at 120 rpm for 72 h at 24°C. The centrifugation of bacterial cultures was carried out at 10,000 rpm for 10 minutes. The pellet was suspended in distilled autoclaved water; optical density to be one was adjusted to 600 nm. The sterilized seeds were then soaked in the bacterial cultures for six hours before sowing.

### Application of chemical fertilizers

Urea and Diammonium Phosphate (DAP) were applied as a source of nitrogen (N) and phosphate (P) fertilizers respectively in three doses i.e. full, half and quarter (Urea 60 Kg ha^-1^ and DAP 30 Kg ha^-1^ as a full dosage, Urea 30 Kg ha^-1^ and DAP 15 Kg ha^-1^ as a half dosage and Urea 15 Kg ha^-1^, DAP 7.5 Kg ha^-1^ as a quarter dosage). Fertilizers were applied by surface broadcast method in which fertilizers were spread uniformly by hand over the entire surface of the ground of the respective treatment. Total quantity of Diammonium Phosphate was applied at the time of sowing while urea was applied at three different stages at an interval of 40 days, however, according to the recommendation; the initial dose of urea was applied at the time of sowing. Two to three irrigations were applied as recommended.

Treatments used in the experiment are enlisted in Table 1.

**Table 1:** 

Treatments	Symbols
Control	**Cont**
Full dosage of NP fertilizers	**CFF**
Half dosage of NP fertilizers	**CFH**
Quarter dosage of NP fertilizers	**CFQ**
*Azospirillum brasilense*	**SP**
*A*. *brasilense*+full dosage of NP fertilizers	**SPF**
*A*. *brasilense*+half dosage NP of fertilizers	**SPH**
*A*. *brasilense*+quarter dosage of NP fertilizers	**SPQ**
*Azotobacter vinelandii*	**BT**
*A*. *vinelandii*+full dosage of NP fertilizers	**BTF**
*A*. *vinelandii*+half dosage of NP fertilizers	**BTH**
*A*. *vinelandii*+quarter dosage of NP fertilizers	**BTQ**

### Chemicals and reagents

*n-*hexane, methanol, boron trifluoride methanol reagent (all chromatography grade), sodium chloride, nonadecanoic acid methyl ester and dichloromethane (DCM) were purchased from Supelco (Sigma–Aldrich) while NaOH was purchased from Merck. Iodine monochloride acetic acid, sodium thiosulphate, potassium iodide, ethanol, diethylether and phenolphthalein were purchased from Sigma–Aldrich. A mixture of 37 FAME components (Supelco, p/n 47885U) was used for the optimisation and validation of the method.

### Oil extraction from seeds

The oil from the seeds of safflower was extracted using Soxhlet apparatus and n-hexane was used as solvent at 60°C for 6 h according to the method of AOCS Ag 1–65 [11].

### Samples preparation and fatty acid analysis

Fatty acid methyl esters were prepared according to the AOCS standard method Ce 2–66 [12].

Separation and quantification of fatty acid methyl esters were carried out using Comprehensive two-dimensional gas chromatography (GC×GC) at Centre of Green Chemistry, Monash University, Melbourne, Australia according to the method described by Nosheen et al. [10].

### Iodine value (gI_2_/100g)

Iodine value was determined according to the method recommended by AOAC [13]. Oil (0.2 g) was taken in 100 mL glass stoppered bottle and dissolved in 15 mL of carbon tetrachloride solution. After addition of 25 mL Wij’s reagent, the contents in the flask were allowed to stand in dark at 25°C for 2 h and then 20 mL of potassium iodide (10%) was added and titrated with sodium thiosulphate (0.2 N) using starch as indicator. The iodine value was calculated using a blank prepared in a similar manner.

### Determination of acid value (mg KOH/g of oil)

Acid value of safflower oil was measured according to the method of Cox and Pearson [14]. Oil sample (0.2 g) was dissolved in 2.5 mL of ethanol:diethylether in the ratio of 1:1 v/v and titrated with NaOH (0.1 N). Phenolphthalein was used as indicator and acid value was calculated.

### Determination of free fatty Acid (%)

Free fatty acid contents were determined according to the following formula

%FreeFattyAcid=0.503×acidvalue

### Determination of refractive index

The refractive index of pure oil and safflower oil biodiesel samples was determined using Abbe 5 refractometer.

### Statistical analysis

Analysis of the experimental data was carried out using Statistix software (version 8.1). Factorial design of Analysis of Variance (ANOVA) (S1 and S2 Files) was used and mean values of respective treatments were compared through Least Significant Difference (LSD) according to Steel and Torrie [15] at P< 0.05. In order to determine the relationship between various parameters, Pearson correlation coefficient test was performed.

## Results

**Fig 1:**
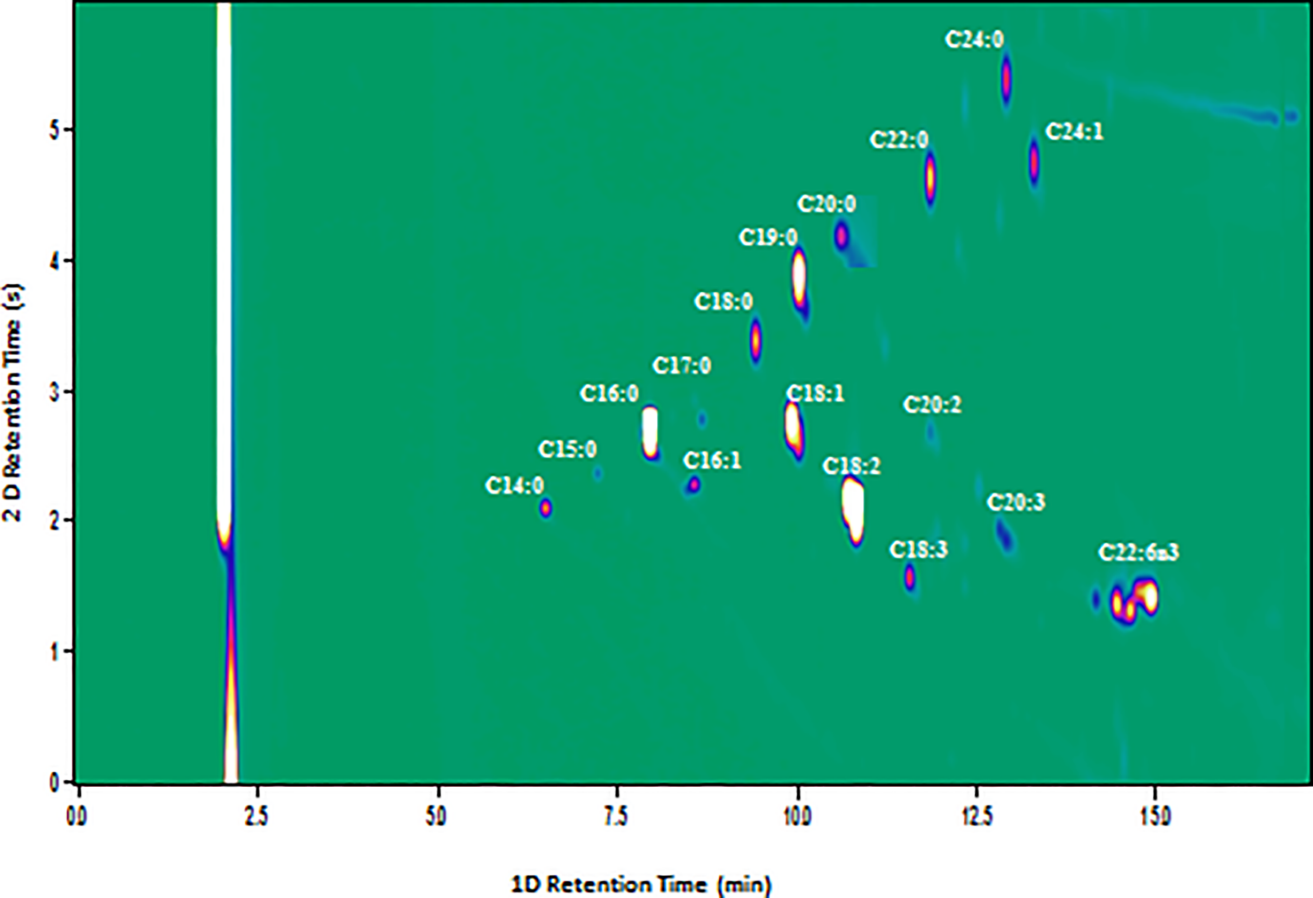
Contour plot showing fatty acid profile of safflower oil analysed by GC×GC-FID (Comprehensive two dimensional gas chromatography equipped with flame ionization detector) on SLB-IL111/IL-59 column set. It showed retention time on one dimensional (minutes) and two dimensional (seconds) column set. C14:0 (Myristic acid), C15:0 (Pentadecylic acid), C16:0 (Palmitic acid), C16:1 (Palmitoleic acid), C17:0 (Margaric acid), C18:0 (Stearic acid), C18:1 (Oleic acid), C18:2 Linoleic acid), C18:3 (Linolenic acid), C19:0 (Nonadecylic acid), C20:0 (Arachidic acid), C20:2 (Eicosadienoic acid), C20:3 (Eicosatrienoic acid), C22:0 (Behenic acid), C22:6n3 (Docosahexaenoic acid), C24:0 (Lingoceric acid), C24:1 (Nervonic acid).

Fig 1 showed the comprehensive two-dimensional chromatograms of safflower which illustrate the fatty acid composition present in the oil extracted from safflower for further analysis. Using dedicated software, individual compounds were visualized as ellipse-shaped peaks in a 2-D contour plot; peak colour and dimension were related to the total analyte quantity. Total 17 fatty acids were detected in safflower oil and among those; five fatty acids (palmitic acid, stearic acid, oleic acid, linolenic acid and linoleic acids) were selected for further study because of their important effect on the quality of biodiesel.

As the data of both years showed non-significant variations for the effect of provided treatments on the already recorded parameters, therefore, the samples of both years were pooled together and then analysis was carried out to assess the effect of treatments on the oil quality of both the varieties of safflower for improved quality biodiesel production.

### Effect of *Azospirillum*, *Azotobacter* and NP fertilizers on fatty acid profile

**Fig 2:**
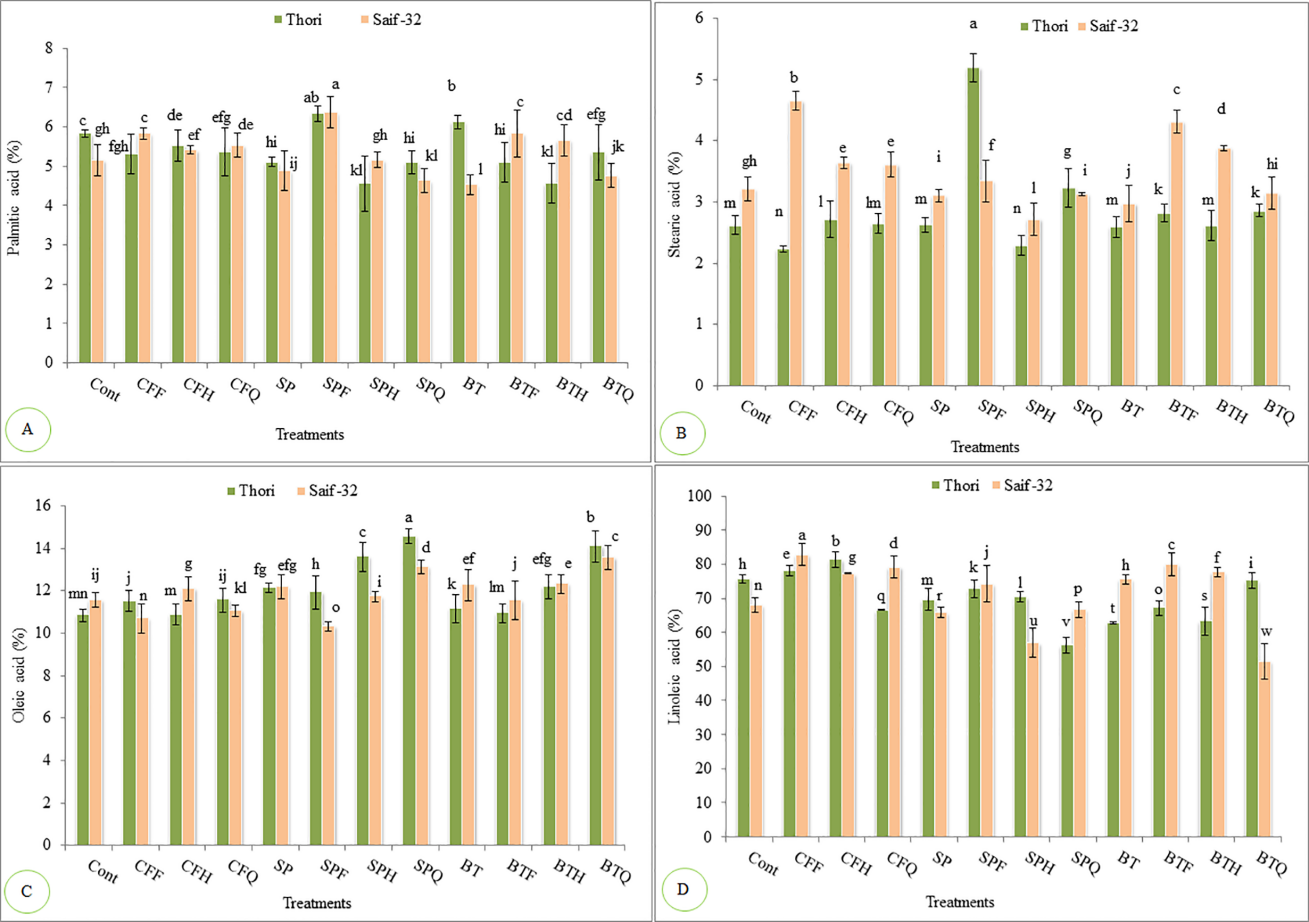
**Effects of *A*. *brasilense*, *A*. *vinelandii* and NP fertilizers on (A) Palmitic acid, (B) Stearic acid, (C) Oleic acid and (D) Linoleic acid of safflower oil.** All such treatments which do not share LSD letters are significantly different from each other while the treatments which share common letters are not significantly different from each other at P<0.05. Detail of treatments. Cont: Control, CFF: NP fertilizers full dosage, CFH: NP fertilizers half dosage, CFQ: NP fertilizers quarter dosage, SP: *A*. *brasilense*, SPF: *A*. *brasilense*+full dosage of NP fertilizers, SPH: *A*. *brasilense*+half dosage of NP fertilizers, SPQ: *A*. *brasilense*+quarter dosage of NP fertilizers, BT: *A*. *vinelandii*, BTF: *A*. *vinelandii*+full dosage of NP fertilizers, BTH *A*. *vinelandii*+half dosage of NP fertilizers, BTQ: *A*. *vinelandii*+quarter dosage of NP fertilizers.

Results showed that all treatments resulted in decrease in the palmitic acid contents of safflower oil except SPF (*Azospirillum* supplemented with full dose of NP fertilizers) (8%) and BT treatments (single inoculation of *Azotobacter*) which resulted in significant increase when compared with the control in case of cultivar Thori (Fig 2A). However, in cultivar Saif-32, SPF treatment resulted in maximum significant increase (23%) in palmitic acid contents compared to that of the control. No significant varietal differences were recorded in term of their effect on palmitic acid contents of safflower oil.

Data regarding stearic acid indicated that SPF treatment resulted in maximum increase of 98% when compared with the control in cultivar Thori (Fig 2B). The treatments CFH, SPQ, BTF and BTQ resulted in significant increase; however rest of the treatments resulted in decrease as compared to the untreated control. In cultivar Saif-32, CFF treatment resulted in maximum significant increase (44%) in stearic acid contents followed by BTF treatment over the control. Varietal differences were observed, cultivar. Thori indicated more increase in stearic acid contents compared to cultivar Saif-32

Almost all the treatments resulted in increase in oleic acid contents in cultivar Thori; however, treatment SPQ (*Azospirillum* supplemented with quarter dose of NP fertilizers) resulted in maximum significant increase (34%) in oleic acid contents compared to the control (Fig 2C). Maximum significant increase (17%) in oleic acid was resulted in BTQ treatment (*Azotobacter* with quarter dose of NP fertilizers) compared to the control in cultivar Saif-32. Single inoculation of *Azospirillum* (SP) and *Azotobacter* (BT) resulted in 5–6% increase in oleic acid contents over the control. The cultivar Thori was more pronounced in increasing the oleic acid contents as compared to cultivar Saif-32.

Linoleic acid contents were significantly decreased by all the treatments except CFF and CFH treatments in cultivar Thori (Fig 2D), however, maximum significant decrease (25%) was recorded in SPQ treatment. In cultivar Saif-32, maximum significant increase (7%) in linoleic acid contents was recorded in CFF treatment compared to that of the control. Maximum significant reduction in linoleic acid contents (24%) was recorded in BTQ treatment in cultivar Saif-32. The cultivar Saif-32 showed more increase in linoleic acid contents as compared to cultivar Thori.

**Fig 3:**
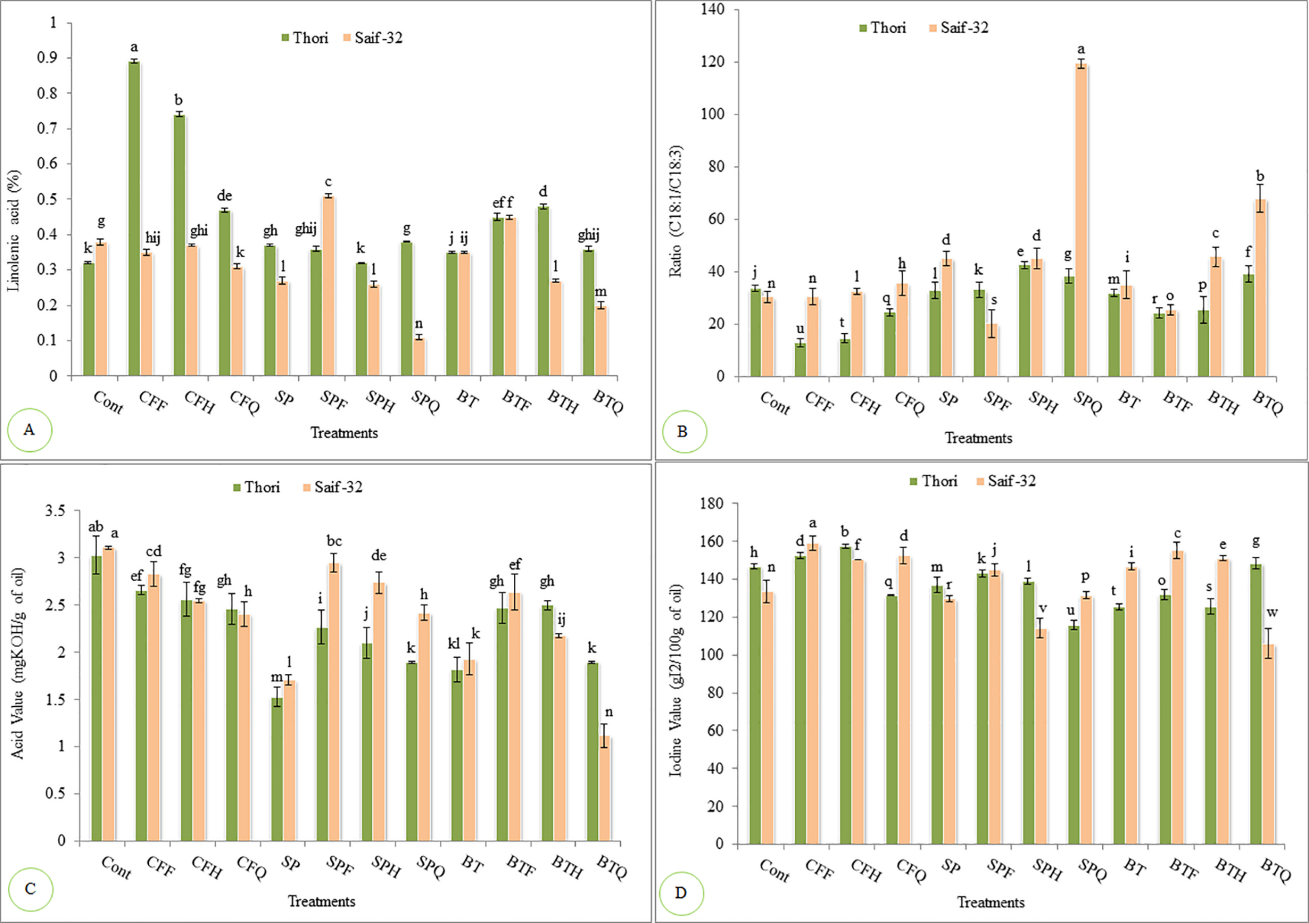
**Effects of *A*. *brasilense*, *A*. *vinelandii* and NP fertilizers on (A) Linolenic Acid, (B) Ratio (C18:1/C18:3), (C) Acid value and (D) Iodine value of safflower oil.** All such treatments which do not share LSD letters are significantly different from each other while the treatments which share common letters are not significantly different from each other at P<0.05. Detail of treatments as described in Fig 2.

Linolenic acid was significantly increased by CFF treatment which resulted in 178% increase compared to the control in cultivar Thori (Fig 3A). In case of cultivar Saif-32, treatment SPF resulted in maximum increase (34%) in linolenic acid contents over control. Treatment BTF also resulted in significant increase, however, all other treatments resulted in reduction of linolenic acid and maximum reduction (71%) was recorded in SPQ treatment when compared with the control. The cultivar Saif-32 was more effective in decreasing the linolenic acid contents as compared to cultivar Thori.

Maximum increase of 25% in the ratio of oleic acid to linolenic acid (C18:1/C18:3) contents was recorded in SPH treatment over control in cultivar Thori (Fig 3B). In cultivar Saif-32, almost all treatments significantly improved the ratio except CFF, SPF and BTF treatments. The maximum significant increase (292%) was resulted in SPQ treatment over control in cultivar Saif-32. The sole inoculation of SP and BT resulted in 48% and 15% significant increase over the control. The cultivar Saif-32 was more dominant in increasing the ratio of oleic acid to linolenic acid as compared to cultivar Thori.

### Effect of *Azospirillum*, *Azotobacter* and NP fertilizers on oil quality of safflower

Fig 3C showed that provided treatments significantly reduced the acid value of safflower oil as compared to that of untreated control in both the varieties. Maximum significant decrease (49%) in acid value was recorded in SP treatment in cultivar Thori. The treatment BTQ resulted in maximum decrease (63%) in acid value in cultivar Saif-32. Single inoculation of SP and BT significantly decreased the acid value as compared to CFF treatment and control. Both the varieties showed almost similar trend for acid value of safflower oil.

Iodine value was significantly reduced by maximum treatments except CFF and CFH treatments which significantly enhanced the iodine value as compared to untreated control in cultivar Thori (Fig 3D). The treatment SPQ resulted in maximum reduction (21%) over control in cultivar Thori. In case of cultivar Saif-32, maximum decrease (20%) in iodine value was recorded in treatment BTQ over the control. Both the varieties of safflower have almost similar tendency to decrease the iodine value of the oil.

**Fig 4:**
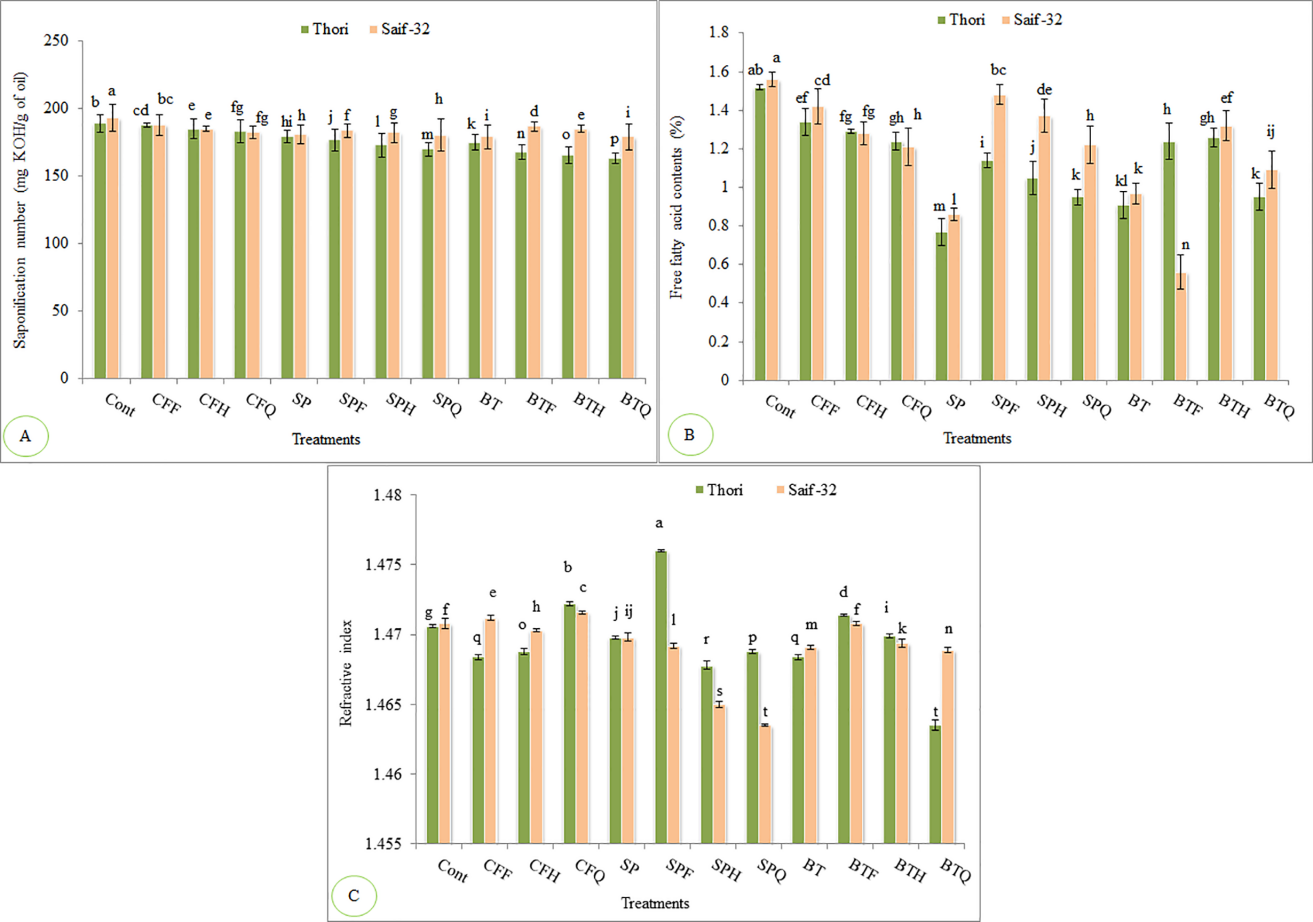
**Effects of *A*. *brasilense*, *A*. *vinelandii* and chemical fertilizers on (A) Saponification number, (B) Free fatty acid contents and (C) Refractive index of safflower oil.** All such treatments which do not share LSD letters are significantly different from each other while the treatments which share common letters are not significantly different from each other at P<0.05. Detail of treatments as described in Fig 2.

All treatments resulted in significantly decrease the saponification number of safflower oil as compared to that of untreated control in both the varieties (Fig 4A). In cultivar Thori, maximum reduction (13%) was recorded in BTQ treatment and similar trend of decrease in saponification number was followed by BTQ treatment in cultivar Saif-32. Both the varieties were equally effective in reducing the saponification number of safflower oil.

All the treatments brought about almost significant reduction in free fatty acid contents of safflower oil in both the varieties (Fig 4B). Treatment SP resulted in maximum decrease (49%) in free fatty acid contents over control in cultivar Thori. In case of cultivar Saif-32, treatment BTF brought about maximum reduction (44%) in free fatty acid contents over the control. The cultivar Thori was more effective in reducing the free fatty acid contents of safflower oil.

Maximum decrease in refractive index of safflower oil was shown by BTQ treatment over the control in cultivar Thori (Fig 4C). While in cultivar Saif-32, maximum decrease in refractive index was resulted in SPQ treatment over the control. The cultivar Thori was more effective in decreasing the refractive index as compared to cultivar Saif-32.

## Discussion

**Fig 5:**
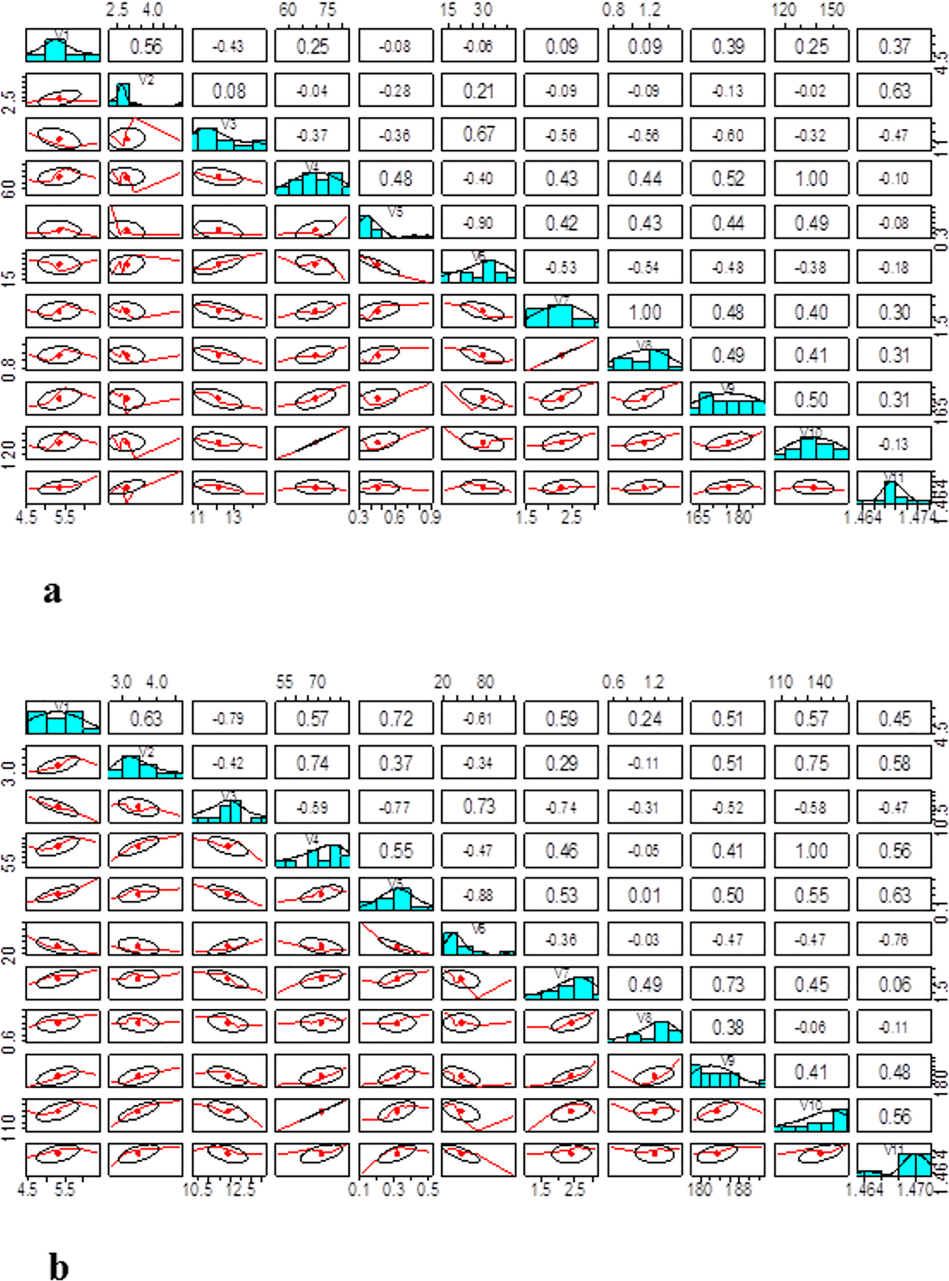
**Correlation between fatty acid and oil quality attributes of a) cv. Thori and b) cv. Saif-32 of safflower.** Detail of the variables is given below V1: Palmitic acid, V2: Stearic acid, V3: Oleic acid, V4: Linoleic acid, V5: Linolenic Acid, V6: Ratio (C18:1/C18:3), V7: Acid value, V8: Free fatty acid, V9: Saponification number, V10: Iodine value, V11: Refractive index.

Fatty acid composition of vegetable oils plays an important role in determining the quality of biodiesel. The present results showed an interesting finding that *Azospirillum* in combination with quarter dose of NP fertilizers (SPQ) gave a considerable increase in oleic acid content. Oleic acid is an important fatty acid to obtain good quality biodiesel production, improving biodiesel properties such as cold flow properties, oxidation stability, specific gravity, cetane number and viscosity. Therefore, supplementing PGPR (Plant Growth Promoting Rhizobacteria) with quarter doses of NP fertilizers would help to improve the quality of oil suitable for biodiesel production. The present findings are in line with those of Dhanasekar and Dhandapani [16] who reported that application of PGPR such as *Azospirillum*, *Azotobacter* and *Rhizobium* in combination with chemical fertilizers significantly influenced the seed yield and oil quality in sunflower. In the present study, a single inoculation of *Azospirillum* and *Azotobacter* also enhanced oleic acid content compared to that of control which is in agreement with the findings of Texier [17] who reported that inoculation of sunflower with PGPR improved the unsaturated/saturated fatty acids ratio and also seed oil content. Similarly, Choudhury and Kennedy [18] reported that inoculation of sunflower with nitrogen fixing bacteria increased the phosphorus level which in turn influenced the seed oil content and the unsaturated/saturated fatty acids ratio. Previously, various scientists [19; 20] reported that the presence of PGPR (specifically nitrogen fixing bacteria) in the rhizosphere and their relationship with the plants improved the nitrogen use efficiency of the plant. Moreover, the production of hormones by the PGPR not only stimulates the development of the roots but also enhanced the nutrient acquisition capability of the plants. These changes impart the alterations in metabolic processes which are responsible for the improved yield and oil quality of the crops. Taken together these facts, the inoculation of PGPB (Plant Growth Promoting Bacteria) reduces the use of fertilizers which are of important environmental concerns of the day. Jha et al. [21] reported that transformation of *Azospirillum brasilense* ACP and its functional expression in *Brassica juncea* not only increased oleic acid and linoleic acid but also resulted in a parallel reduction of C22:1. Our results indicated that half dose of NP fertilizers resulted in improving the fatty acid composition in terms of oleic acid which confirm the findings of Texier [17] who demonstrated that in oil seed crops, during the process of seed development, the fatty acid composition is under genetic control and the process of fatty acid biosynthesis requires the C and N skeleton and thus nitrogen fertilizers play an important role in modifying the fatty acid composition of oil seed crops. Di Benedetto et al. [22] reported that the PGPB isolated from the soil of South Italy showed improved nutrient and nitrogen use efficiency of the wheat crop. This nutrient/nitrogen use efficiency of the crops is an important factor for the metabolic processes which provide nutrients (C, N) for the biosynthesis of fatty acids. Hence, the nitrogen use efficiency play important role in alteration of fatty acid composition of the respective crop. Gopinath et al. [23] reported that fatty acid methyl esters significantly influenced the cetane number of biodiesel, which increases with increase in chain length and decreases with degree of unsaturation. In the present study, the ratio of monounsaturated (oleic acid)/polyunsaturated (linolenic acid) was significantly increased by the SPQ treatment which plays important role in the improvement of the oil quality and oxidation stability with perspective to biodiesel production. Polyunsaturated fatty acids are more prone to oxidation and lead to poor storage properties of biodiesel as compared to monounsaturated fatty acids [24]. Fig 5A and 5B showed that there is a negative correlation between monounsaturated (oleic acid) and polyunsaturated (linolenic acid) which leads toward the improvement of oxidation stability of the oil and it is a very important finding of the study.

Application of PGPR in combination with quarter dose of NP fertilizers markedly improved the oil quality of safflower by improving the oxidation stability, cetane number, viscosity and cold flow properties which are very important variables affecting biodiesel quality.

For good quality biodiesel production, the properties of oil play an important role. Acid value is an important variable for monitoring the quality of biodiesel during transeterification reaction and storage. According to the present study, maximum reduction in acid value was resulted by BTQ treatment in cv. Saif-32. These findings are in parallel to that of Abd El-Gawad et al. [25] who reported that *Azotobacter chroococcum and Bacillus megaterium* reduced the acid value and peroxide value of canola as compared to the control. Similar findings were reported by Nosheen et al. [26] that acid value, free fatty acid contents and specific gravity of canola oil were significantly reduced by the application of *Azospirillum* and chemical fertilizers treatments. The reduction in the above mentioned parameters lead towards the decrease in engine corrosion, rancidity and density of oil; hence, indirectly improved the quality of oil for biodiesel production and also reduce the use of chemical fertilizers. Dodos et al. [27] reported that due to increase in acid value of vegetable oil, formation of shorter fatty acid and acidic secondary oxidation products occurred which leads to the corrosion of engine [24]. The decrease in acid value might be due to the production of phytohormone by PGPR as Ullah and Bano [28] reported that application of cytokinin decreased the acid value of safflower oil. The lower acidity of vegetable oil and biodiesel leads to a considerable increase in oxidation stability and improved storage properties of the fuel [29]. Results in the current study indicated that treatment SP resulted in maximum reduction of free fatty acid content of oil followed by BT, BTF and BTQ treatments respectively. It is reported that the yield of biodiesel is highly influenced by the presence of free fatty acid in the oil during base catalysed transesterification reaction [30]. The presence of free fatty acid contents in higher amount resulted in soap formation and ultimately leading to the reduction of biodiesel yield and quality [31].

Saponification number is an important variable that affects the quality of biodiesel. Our results indicated a reduction in saponification value especially with the BTQ treatment. Saponification is negatively correlated with the degree of unsaturation [23]. It was reported in the previous study that application of nitrogen fertilizer at the rate of 108–216 kg per hectare to the plant reduced the saponifiable number and refractive index and lead to parallel increase in unsaponifiable number [32]; however no literature was found for the effect of PGPR on oil saponification number.

Refractive index and Iodine value are important factors strongly correlated with each other (Fig 5A and 5B) and with degree of unsaturation of fatty acids. Polyunsaturated fatty acid has higher iodine values and refractive indexes as compared to monounsaturated fatty acid [33]. The present work showed that maximum reduction in both the variables was resulted in SPQ treatment. These results are in line with the findings of Lawania et al. [34] who reported that the application of nitrogen fertilizers in combination with biofertilizers (*Azotobacter*) reduced the iodine value of linseed oil. Reduced values of iodine and refractive indices improve the quality and oxidation stability of oil [35]. There is a negative correlation between oleic acid content and other oil quality parameters such as acid value, free fatty acid, iodine value, refractive index etc. It is an important finding of the study because increase in oleic acid and in parallel decrease in acid value, free fatty acid, iodine value, refractive index etc. imparts good quality biodiesel production (Fig 5A and 5B) by improving the oxidation stability and improving other properties of biodiesel. The higher iodine value tends to result in deposit formation hence causes problems of storage stability. Iodine value decrease with decrease in refractive index and improves the quality and oxidation stability of oil [35]. Ahmad et al. [36] reported that impregnation of PGPB with urea and DAP improved the nitrogen and phosphorus use efficiency by the wheat plant and also improved the photosynthetic rate and yield. The PGPB such as *Azospirillum* improved the nitrogen use efficiency by biological nitrogen fixation and facilitate the uptake of nutrient by the plants. As the treatments containing PGPB with reduced amount of fertilizers (half or quarter doses) are efficient in improving the oil quality, as a result these treatments reduced the application of chemical fertilizers which is the important finding of the study and leads towards the ecofriendly sustainable agriculture.

## Conclusion

It can be concluded from the above results and discussion that oil quality parameters such as fatty acid profile and oil properties were significantly improved by the inoculation of PGPR supplemented with quarter and half rates of NP fertilizers. Oleic acid content and C18:1/C18:3 ratios were significantly improved by *Azospirillum* in combination with half rate of NP fertilizers. However, oil properties such as acid value, iodine value, refractive index etc. were significantly improved by BTQ and SPQ treatments in both the varieties. The behaviour of the varieties varies in response to provided treatments for the specific parameter. According to these results we can reduced the use of NP fertilizers upto 50%–75% with the application of *Azospirillum* and *Azotobacter* for green, sustainable and environment friendly good quality biodiesel production. However, further research at molecular level is needed to confirm the interaction of PGPR with fatty acid biosynthesis pathway. In order to achieve the goal of better safflower oil quality production, the exploration of molecular pathways in combination with biochemical and genetic approaches which are linked with the current study are needed.

## Supporting information

S1 File Minimal data. (XLSX)

S2 File ANOVA Tables_Supplemental file. (DOCX)
