# Supplementary material for: Improvement of safflower oil quality for biodiesel production by integrated application of PGPR under reduced amount of NP fertilizers
Source: PLoS One. 2018 Aug 10;13(8):e0201738. doi: 10.1371/journal.pone.0201738 (PMC6086401; doi:10.1371/journal.pone.0201738)
Supplement: S2 File — (DOCX) [file pone.0201738.s002.docx]

**ANNOVA Tables**

**Analysis of Variance Table for palmitic acid (C16:0)**

**Source DF SS MS F P**

replicat 2 0.0261 0.01307

Treat 11 11.0702 1.00638 53.75 0.0000

var 1 0.0304 0.03042 1.62 0.2088

Treat*var 11 9.0497 0.82270 43.94 0.0000

Error 46 0.8612 0.01872

Total 71 21.0377

Grand Mean 5.3364 CV 2.56

**Analysis of Variance Table for stearic acid (C18:0)**

**Source DF SS MS F P**

replicat 2 0.0091 0.00454

Treat 11 13.3530 1.21391 592.48 0.0000

var 1 6.6066 6.60661 3224.54 0.0000

Treat*var 11 17.2698 1.56999 766.28 0.0000

Error 46 0.0942 0.00205

Total 71 37.3328

Grand Mean 3.1757 CV 1.43

**Analysis of Variance Table for Oleic acid (C18:1)**

**Source DF SS MS F P**

replicat 2 0.0210 0.01051

Treat 11 65.3730 5.94300 615.27 0.0000

var 1 1.1375 1.13753 117.77 0.0000

Treat*var 11 18.3628 1.66934 172.82 0.0000

Error 46 0.4443 0.00966

Total 71 85.3387

Grand Mean 12.002 CV 0.82

**Analysis of Variance Table for linoleic acid (C18:2)**

**Source DF SS MS F P**

replicat 2 0.06 0.030

Treat 11 2321.81 211.074 8832.89 0.0000

var 1 166.47 166.470 6966.36 0.0000

Treat*var 11 1853.74 168.521 7052.19 0.0000

Error 46 1.10 0.024

Total 71 4343.17

Grand Mean 71.473 CV 0.22

**Analysis of Variance Table for linolenic acid (C18:3)**

**Source DF SS MS F P**

replicat 2 0.00050 0.00025

Treat 11 0.82282 0.07480 414.70 0.0000

var 1 0.34031 0.34031 1886.70 0.0000

Treat*var 11 0.60800 0.05527 306.44 0.0000

Error 46 0.00830 0.00018

Total 71 1.77993

Grand Mean 0.3915 CV 3.43

**Analysis of Variance Table for Ratio of C18:1/C18:3**

**Source DF SS MS F P**

replicat 2 40.5 20.26

Treat 11 14751.7 1341.07 153.06 0.0000

var 1 3845.8 3845.81 438.94 0.0000

Treat*var 11 9456.3 859.67 98.12 0.0000

Error 46 403.0 8.76

Total 71 28497.4

Grand Mean 36.298 CV 8.15

**Analysis of Variance Table for Acid value**

**Source DF SS MS F P**

rep 2 0.0182 0.00908

treat 11 13.9777 1.27070 229.32 0.0000

var 1 0.2392 0.23920 43.17 0.0000

treat*var 11 2.7094 0.24631 44.45 0.0000

Error 46 0.2549 0.00554

Total 71 17.1993

**Analysis of Variance Table for Iodine value**

**Source DF SS MS F P**

replicat 2 0.1 0.049

Treat 11 7954.2 723.113 7769.96 0.0000

var 1 56.1 56.111 602.93 0.0000

Treat*var 11 7847.4 713.398 7665.57 0.0000

Error 46 4.3 0.093

Total 71 15862.1

Grand Mean 138.76 CV 0.22

**Analysis of Variance Table for Free fatty acid contents**

**Source DF SS MS F P**

rep 2 0.00460 0.00230

treat 11 3.09174 0.28107 200.48 0.0000

var 1 0.06052 0.06052 43.17 0.0000

treat*var 11 1.13024 0.10275 73.29 0.0000

Error 46 0.06449 0.00140

Total 71 4.35159

Grand Mean 1.1712 CV 3.20

**Analysis of Variance Table for Saponification number**

**Source DF SS MS F P**

rep 2 1.36 0.681

treat 11 2183.61 198.510 508.09 0.0000

var 1 997.56 997.556 2553.25 0.0000

treat*var 11 874.78 79.525 203.55 0.0000

Error 46 17.97 0.391

Total 71 4075.28

**Analysis of Variance Table for Refractive Index**

**Source DF SS MS F P**

teat 11 3.101E-04 2.819E-05 44126.3 0.0000

var 1 2.765E-06 2.765E-06 4328.09 0.0000

teat*var 11 1.805E-04 1.641E-05 25682.3 0.0000

Error 48 3.067E-08 6.389E-10

Total 71 4.934E-04

Grand Mean 1.4694 CV 0.00
